# Impact of laparoscopic surgery on short‐term and long‐term outcomes in elderly obese patients with colon cancer

**DOI:** 10.1002/ags3.12678

**Published:** 2023-04-23

**Authors:** Nobuaki Hoshino, Koya Hida, Yusuke Fujita, Masaichi Ohira, Heita Ozawa, Hiroyuki Bando, Tomonori Akagi, Yohei Kono, Kentaro Nakajima, Yutaka Kojima, Takatoshi Nakamura, Masafumi Inomata, Seiichiro Yamamoto, Yoshiharu Sakai, Takeshi Naitoh, Masahiko Watanabe, Kazutaka Obama

**Affiliations:** ^1^ Department of Surgery Kyoto University Graduate School of Medicine Kyoto Japan; ^2^ Department of Surgery Baba Memorial Hospital Sakai Japan; ^3^ Department of Colorectal Surgery Tochigi Cancer Center Utsunomiya Japan; ^4^ Department of Gastroenterological Surgery Ishikawa Prefectural Central Hospital Kanazawa Japan; ^5^ Department of Gastroenterological and Pediatric Surgery Oita University Faculty of Medicine Yufu Japan; ^6^ Department of Surgery NTT Medical Center Shinagawa‐ku Japan; ^7^ Department of Coloproctological Surgery Juntendo University Faculty of Medicine Bunkyo‐ku Japan; ^8^ Department of Surgical Oncology Dokkyo Medical University Graduate School of Medicine Shimotsuga‐gun Japan; ^9^ Department of Gastroenterological Surgery Tokai University School of Medicine Shibuya City Japan; ^10^ Japanese Red Cross Osaka Hospital Osaka Japan; ^11^ Department of Lower Gastrointestinal Surgery Kitasato University School of Medicine Sagamihara Japan; ^12^ Kitasato University Kitasato Institute Hospital Minato City Japan

**Keywords:** colon neoplasms, complication, elderly, obese, prognosis

## Abstract

**Background:**

Laparoscopic surgery is reported to be useful in obese or elderly patients with colon cancer, who are at increased risk of postoperative complications because of comorbidities and physical decline. However, its usefulness is less clear in patients who are both elderly and obese and may be at high risk of complications.

**Methods:**

Data for obese patients (body mass index ≥25) who underwent laparoscopic or open surgery for stage II or III colon cancer between January 2009 and December 2013 were collected by the Japan Society of Laparoscopic Colorectal Surgery. Surgical outcomes, postoperative complications, and relapse‐free survival (RFS) were compared between patients who underwent open surgery and those who underwent laparoscopic surgery according to whether they were elderly (≥70 y) or nonelderly (<70 y).

**Results:**

Data of 1549 patients (elderly, *n* = 598; nonelderly, *n* = 951) satisfied the selection criteria for analysis. Length of stay was shorter and surgical wound infection was less common in elderly obese patients who underwent laparoscopic surgery than in those underwent open surgery. There were no significant between‐group differences in overall complications, anastomotic leakage, ileus/small bowel obstruction, or RFS. There were also no significant differences in RFS after laparoscopic surgery according to patient age.

**Conclusion:**

Laparoscopic surgery is safe in elderly obese patients with colon cancer and does not worsen their prognosis. There was no significant difference in the effectiveness of laparoscopic surgery between obese patients who were elderly and those who were nonelderly.

## Graphical abstract

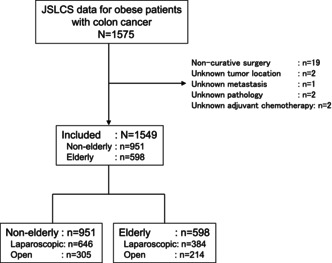

## 1 INTRODUCTION

Since laparoscopic surgery was first reported in the 1990s, it has been demonstrated to be effective in colon cancer and is now widely performed.^1^ Compared with open surgery, laparoscopic surgery has several advantages, including a magnification effect, which allows recognition of microanatomy and more precise surgery, and smaller surgical wounds, which shortens the recovery time after surgery, despite often needing a longer operation time.^2^ However, laparoscopic surgery also has some disadvantages, particularly in obese patients. These drawbacks include a negative impact of insufflation on acid–base balance, including an increased PaCO_2_ and decreased base excess in arterial blood; a negative effect on hemodynamics by increasing heart rate and mean blood pressure as well as decreasing blood flow in the portal and femoral veins; and reduction of cardiac function by decreasing stroke volume and increasing systemic vascular resistance.^3^, ^4^ The impact of insufflation in the elderly, in whom organ function is generally reduced, has been a matter of debate. Nevertheless, laparoscopic surgery has been demonstrated to be safe and effective when performed for colon cancer in obese patients and in the elderly.^5^, ^6^ However, there are few reports on laparoscopic surgery in patients who are both obese and elderly, who are considered to be at particularly high surgical risk. In this study, we compared the short‐term and long‐term postoperative outcomes of laparoscopic surgery for colon cancer in obese patients according to whether they were elderly or nonelderly.

## 2 PATIENTS AND METHODS

### 2.1 Study design and setting

Data for obese patients (body mass index [BMI] ≥25) who underwent laparoscopic or open surgery for stage II or III colon cancer between January 2009 and December 2013 were collected by the Japan Society of Laparoscopic Colorectal Surgery from 46 participating hospitals in the laparoscopic versus open surgery for obesity study (the LOVERY study).^7^ Patients who had undergone nonradical surgery and those for whom information on tumor location, metastasis, differentiation, and adjuvant chemotherapy were missing were excluded. Finally, patients who had undergone curative surgery for colon cancer and for whom sufficient information was available to investigate their prognosis were included. The study was approved by the Ethics Committee of Kyoto University (approval number R1728). The need for informed consent was waived in view of the anonymity of the data.

### 2.2 Statistical analysis

Categorical variables are shown as the number and percentage and were compared between groups using Fisher's exact test. Continuous variables are summarized as the mean and standard deviation and were compared using the *t*‐test. Surgical outcomes, postoperative complications, and relapse‐free survival (RFS) were compared between patients who underwent laparoscopic surgery and those who underwent open surgery. RFS was defined as the interval between the date of surgery and the date of recurrence or death, whichever came first, and investigated in univariable and multivariable Cox regression models. Clinically and statistically significant factors were considered as confounders in the multivariable model. Survival curves for RFS were estimated by the Kaplan–Meier method and tested with the log‐rank test. All comparisons between laparoscopic surgery and open surgery were performed separately for patients who were elderly (aged ≥70 y) and those who were nonelderly (aged <70 y). All statistical analyses were performed using JMP statistical software v. 15 (SAS Institute, Cary, NC, USA). All *P*‐values were two‐sided and considered statistically significant when <0.05.

## 3 RESULTS

### 3.1 Patient characteristics

**FIGURE 1:**
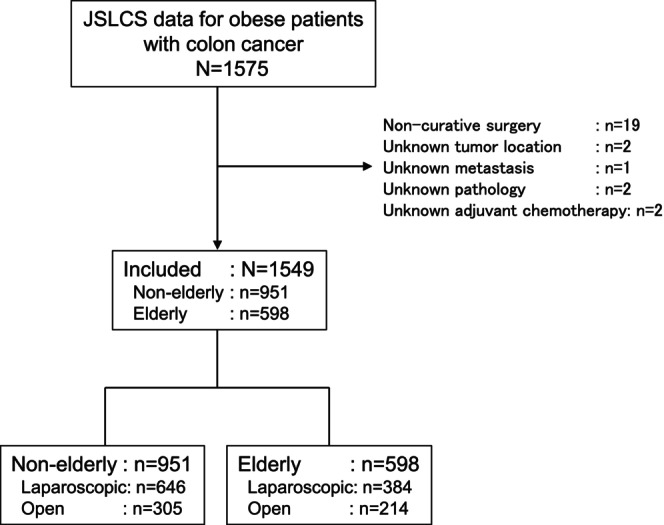
Flow diagram showing the patient selection process. JSLCS, Japan Society of Laparoscopic Colorectal Surgery.

**FIGURE 2:**
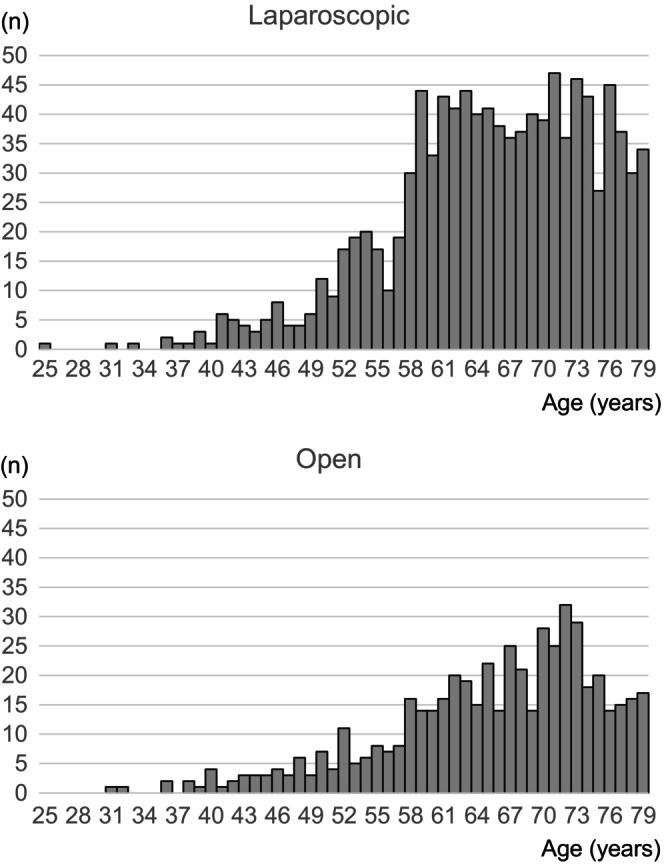
Distribution of patient age according to whether surgery was open or laparoscopic.

Data were available for 1575 obese patients with colon cancer, 1549 of whom satisfied the study inclusion criteria (elderly, *n* = 598; nonelderly, *n* = 951; Figure 1). Distributions of BMI in elderly and nonelderly patients are shown in Figure S1. The patient characteristics are shown in Table 1 and the age distribution according to surgical approach is shown in Figure 2. There was no significant difference in BMI between patients who underwent laparoscopic surgery and those who underwent open surgery regardless of age. Hypertension and diabetes were more common in elderly patients than in nonelderly patients, but were similar between laparoscopic surgery and open surgery irrespective of age. Cardiovascular and respiratory diseases were more common in the elderly than in the nonelderly and were more common in patients who underwent open surgery than in those who underwent laparoscopic surgery regardless of age. Patients with more advanced cancer were more likely to undergo open surgery whether or not they were elderly. Adjuvant chemotherapy was more likely to be administered in nonelderly patients than in elderly patients.

**TABLE 1:** Patient characteristics.

Variable	Category	Nonelderly				Elderly			
		Laparoscopic		Open		Laparoscopic		Open	
		*n*	%	*n*	%	*n*	%	*n*	%
Sex	Female	224	34.7	126	41.3	169	44.0	96	44.9
	Male	422	65.3	179	58.7	215	56.0	118	55.1
BMI	Mean (SD)	27.7	(2.8)	27.7	(2.6)	27.3	(2.1)	27.2	(2.1)
ASA PS	1	222	34.4	113	37.0	101	26.3	43	20.1
	2	424	65.6	192	63.0	283	73.7	171	79.9
HT	−	365	56.5	176	57.7	156	40.6	86	40.2
	+	281	43.5	129	42.3	228	59.4	128	59.8
DM	−	489	75.7	232	76.1	274	71.4	155	72.4
	+	157	24.3	73	23.9	110	28.6	59	27.6
CVD	−	620	96.0	288	94.4	355	92.4	191	89.3
	+	26	4.0	17	5.6	29	7.6	23	10.7
RD	−	614	95.0	281	92.1	357	93.0	194	90.7
	+	32	5.0	24	7.9	27	7.0	20	9.3
CD	−	578	89.5	276	90.5	328	85.4	170	79.4
	+	68	10.5	29	9.5	56	14.6	44	20.6
Tumor location	LC	429	66.4	191	62.6	222	57.8	112	52.3
	RC	217	33.6	114	37.4	162	42.2	102	47.7
pT	0–2	91	14.1	20	6.6	38	9.9	14	6.5
	3,4	555	85.9	285	93.4	346	90.1	200	93.5
pN	−	322	49.8	156	51.1	214	55.7	111	51.9
	+	324	50.2	149	48.9	170	44.3	103	48.1
Differentiation	Differentiated	598	92.6	270	88.5	358	93.2	199	93.0
	Undifferentiated	34	5.3	30	9.8	20	5.2	14	6.5
	Other	14	2.2	5	1.6	6	1.6	1	0.5
Adjuvant chemotherapy	−	301	46.6	128	42.0	229	59.6	126	58.9
	+	345	53.4	177	58.0	155	40.4	88	41.1

Abbreviations: ASA PS, American Society of Anesthesiologists performance status; BMI, body mass index; CD, cerebral disease; CVD, cardiovascular disease; DM, diabetes mellitus; HT, hypertension; LC, left colon; RC, right colon; RD, respiratory disease; SD, standard deviation.

### 3.2 Surgical outcomes

Operation time was significantly longer, estimated blood loss was significantly smaller, and combined resection of other organs was significantly less common in patients who underwent laparoscopic surgery than in those who underwent open surgery regardless of age. In nonelderly patients, the number of lymph nodes examined and the number of lymph node metastases were significantly smaller in patients who underwent laparoscopic surgery than in those who underwent open surgery, whereas there were no significant differences in elderly patients. There was no significant difference in length of hospital stay between nonelderly patients who underwent laparoscopic surgery and nonelderly patients who underwent open surgery; however, length of stay was significantly shorter in elderly patients who underwent laparoscopic surgery than in elderly patients who underwent open surgery (Table 2).

**TABLE 2:** Surgical outcomes.

	Nonelderly					Elderly				
	Laparoscopic		Open		*P*‐value	Laparoscopic		Open		*P*‐value
	Mean	SD	Mean	SD		Mean	SD	Mean	SD	
Operation time, min	240	95	206	68	<0.001	239	79	199	68	<0.001
Estimated blood loss, mL	63	142	232	281	<0.001	83	172	223	307	<0.001
Combined resection of other organs^a^(‐/+)	625	96.7	267	87.5	<0.001	378	98.4	191	89.3	<0.001
	21	3.3	38	12.5		6	1.6	23	10.7	
NLNE	21.8	12.4	24.3	17.6	0.014	20.0	10.3	21.1	13.6	0.242
NLNM	1.4	2.5	1.9	3.9	0.034	1.2	2.4	1.2	2.1	0.760
Length of stay, days	13.8	36.9	14.4	9.4	0.757	14.0	11.2	17.4	27.0	0.033

Abbreviations: NLNE, number of lymph node examined; NLNM, number of lymph node metastasis; SD, standard deviation.

^a^ Number, percentage.

There was no significant difference in the proportion of patients in whom laparoscopic surgery was converted to open surgery between elderly patients and nonelderly patients. The reasons for conversion are shown in Table 3. The proportion of tumors that progressed was high regardless of age. Poor visibility of the surgical field was more common in nonelderly patients and adhesions and bleeding were more common in elderly patients.

**TABLE 3:** Conversion to open surgery.

	Nonelderly *n* = 646		Elderly *n* = 384		*P*‐value
	*n*	%	*n*	%	
Conversion	24	3.7	20	5.2	0.267
Reason for conversion					
Adhesion	3	12.5	6	30.0	
Bleeding	2	8.3	5	25.0	
Other organ damage	1	4.2	0	0.0	
Tumor progression	5	20.8	7	35.0	
Poor visibility	8	33.3	2	10.0	
Other	5	20.8	0	0.0	

### 3.3 Postoperative complications (Clavien–Dindo grade ≥2)

Overall complications and ileus/small bowel obstruction were less common and anastomotic leakage was more common after laparoscopic surgery than after open surgery in both age groups, although these differences were not statistically significant. However, surgical wound infection was significantly less common in elderly patients who underwent laparoscopic surgery than in elderly patients who underwent open surgery (Table 4).

**TABLE 4:** Postoperative complications (Clavien−Dindo grade ≥2).

	Nonelderly					Elderly				
	Laparoscopic		Open		*P*‐value	Laparoscopic		Open		*P*‐value
	*n*	%	*n*	%		*n*	%	*n*	%	
Overall complications (‐/+)	583	90.2	268	87.9	0.260	330	85.9	179	83.6	0.473
	63	9.8	37	12.1		54	14.1	35	16.4	
Anastomotic leakage (‐/+)	624	96.6	299	98.0	0.304	367	95.6	209	97.7	0.258
	22	3.4	6	2.0		17	4.4	5	2.3	
Ileus/small bowel obstruction (‐/+)	636	98.5	294	96.4	0.057	371	96.6	201	93.9	0.144
	10	1.5	11	3.6		13	3.4	13	6.1	
Surgical wound infection (‐/+)	636	98.5	295	96.7	0.093	379	98.7	204	95.3	0.025
	10	1.5	10	3.3		5	1.3	10	4.7	

### 3.4 Relapse‐free survival

**FIGURE 3:**
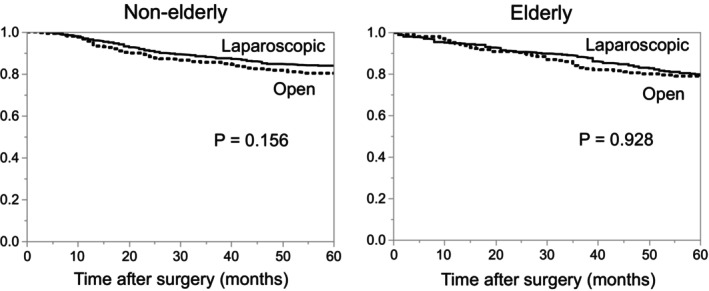
Relapse‐free survival curves for laparoscopic and open surgeries in elderly and nonelderly patients.

In univariable analysis, there was no significant difference in RFS between patients who underwent laparoscopic surgery and those who underwent open surgery according to whether they were nonelderly (unadjusted hazard ratio [HR] 0.80, 95% confidence interval [CI] 0.58–1.10, *P* = 0.164) or elderly (unadjusted HR 0.97, 95% CI 0.67–1.41, *P* = 0.884). Survival curves for RFS in laparoscopic and open surgeries are presented in Figure 3. Sex, pT, pN, differentiation, and adjuvant chemotherapy were identified as significant factors in the multivariable model. After adjusting for these factors, there was still no significant difference in RFS between laparoscopic surgery and open surgery in either nonelderly patients (adjusted HR 0.82, 95% CI 0.60–1.14, *P* = 0.238) or elderly patients (adjusted HR 1.02, 95% CI 0.70–1.49, *P* = 0.914, Table 5). There was also no significant age‐related difference in the impact of laparoscopic surgery on RFS (*P* for interaction, 0.324).

**TABLE 5:** Risk factors for relapse‐free survival.

Patients	Variable	Category	Univariable analysis			Multivariable analysis		
			HR	95% CI	*P*‐value	HR	95% CI	*P*‐value
Nonelderly	Sex	Female/Male	0.78	0.56–1.08	0.136	0.72	0.51–1.00	0.049
	BMI	per unit	0.98	0.92–1.04	0.602			
	ASA‐PS	2/1	0.90	0.66–1.24	0.900			
	HT	+/−	1.00	0.73–1.36	0.978			
	DM	+/−	0.98	0.68–1.40	0.905			
	CVD	+/−	0.39	0.12–1.23	0.107			
	RD	+/−	0.77	0.38–1.57	0.477			
	CD	+/−	1.00	0.61–1.65	1.000			
	Approach	Laparoscopy/Open	0.80	0.58–1.10	0.164	0.82	0.60–1.14	0.238
	Tumor location	LC/RC	1.02	0.74–1.41	0.914			
	pT	3–4/0–2	1.73	0.96–3.11	0.069	2.84	1.56–5.19	0.001
	pN	+/−	2.66	1.89–3.73	<0.001	3.52	2.24–5.54	<0.001
	Differentiation	Differentiated	Ref			Ref		
		Undifferentiated	0.88	0.46–1.66	0.688	0.76	0.40–1.45	0.414
		Other	0.94	0.30–2.94	0.912	1.20	0.38–3.76	0.761
	AC	+/−	1.79	1.29–2.49	<0.001	0.88	0.57–1.35	0.553
Elderly	Sex	Female/Male	0.68	0.47–0.99	0.042	0.66	0.45–0.95	0.028
	BMI	per unit	1.02	0.93–1.10	0.638			
	ASA PS	2/1	0.87	0.58–1.30	0.488			
	HT	+/−	0.78	0.54–1.11	0.170			
	DM	+/−	1.01	0.68–1.50	0.964			
	CVD	+/−	1.17	0.65–2.13	0.602			
	RD	+/−	1.41	0.78–2.56	0.257			
	CD	+/−	1.00	0.62–1.62	0.994			
	Approach	Laparoscopy/Open	0.97	0.67–1.41	0.884	1.02	0.70–1.49	0.914
	Tumor location	LC/RC	0.96	0.67–1.38	0.841			
	pT	3–4/0–2	1.14	0.58–2.25	0.709	1.50	0.75–3.03	0.253
	pN	+/−	1.78	1.23–2.55	0.002	2.49	1.58–3.93	<0.001
	Differentiation	Differentiated	Ref			Ref		
		Undifferentiated	1.36	0.69–2.69	0.371	1.57	0.79–3.12	0.194
		Other	1.62	0.40–6.55	0.501	1.40	0.34–5.74	0.642
	AC	+/−	1.11	0.77–1.59	0.574	0.64	0.41–1.00	0.050

Abbreviations: AC, adjuvant chemotherapy; ASA PS, American Society of Anesthesiologists performance status; BMI, body mass index; CD, cerebral disease; CI, confidence interval; CVD, cardiovascular disease; DM, diabetes mellitus; HR, hazard ratio; HT, hypertension; LC, left colon; RC, right colon; RD, respiratory disease.

## 4 DISCUSSION

The findings of this study suggest that laparoscopic surgery for colon cancer may be more beneficial in elderly obese patients than in nonelderly obese patients in terms of surgical outcomes, including length of hospital stay, number of lymph nodes examined, number of lymph node metastases, and surgical wound infection. Also, no difference in the long‐term outcomes of laparoscopic surgery was found between elderly and nonelderly patients.

Obesity is closely associated with hypertension, diabetes, and heart disease, all of which increase the risk of postoperative complications. Furthermore, the high amount of intra‐abdominal fat and narrow intra‐abdominal space increases the difficulty of laparoscopic surgery, and operations tend to be lengthy.^8^, ^9^ Operative time becomes longer with increasing BMI, and obesity has been reported to be a risk factor for postoperative complications and a poor prognosis after laparoscopic surgery.^10^, ^11^, ^12^ However, there is an increasing body of evidence showing that the minimally invasive nature of laparoscopic surgery allows for early postoperative recovery in obese patients.^13^, ^14^, ^15^, ^16^, ^17^, ^18^, ^19^ The usefulness of laparoscopic surgery for colon cancer in obese patients has been the subject of much debate, and there are two main ways in which it has been studied: (1) comparison of obese and nonobese patients with colon cancer undergoing laparoscopic surgery, and (2) comparison of laparoscopic surgery with open surgery in obese patients with colon cancer. In laparoscopic surgery for colon cancer, the operation time was found to be longer and the rate of conversion to open surgery to be higher in obese patients than in nonobese patients, but the postoperative recovery and length of hospital stay were similar and laparoscopic surgery was reported to be safe and useful for obese patients.^13^, ^14^, ^15^, ^16^ Also, laparoscopic surgery in obese patients with colon cancer had a longer operation time compared with open surgery, but had less blood loss, no increase in postoperative complications or need for reoperation, and a shorter hospital stay.^17^, ^18^, ^19^

Elderly patients often have comorbidities and an age‐related decline in organ function, and the usefulness of laparoscopic surgery for colon cancer in these patients has also been the subject of much debate.^20^, ^21^, ^22^ Again, this has been investigated in two ways: (1) comparison of nonelderly and elderly patients with colon cancer undergoing laparoscopic surgery, and (2) comparison of laparoscopic surgery with open surgery in elderly patients with colon cancer. A study that compared nonelderly and elderly patients who underwent laparoscopic surgery for colon cancer found a similar operation time, amount of blood loss, rate of conversion to open surgery, postoperative complication rate, and operative quality based on histopathological results.^5^, ^23^, ^24^ Furthermore, there was no difference in long‐term outcomes between the two groups, and aging itself was not identified to increase the risk in patients undergoing laparoscopic surgery for colon cancer.^23^, ^24^ In elderly patients with colon cancer, laparoscopic surgery was reported to need a longer operation time compared with open surgery, but resulted in less blood loss, fewer postoperative complications, and comparable operative quality.^25^, ^26^, ^27^, ^28^ Moreover, there was no difference in the prognosis according to whether surgery was laparoscopic or open.^29^

Although there have been many reports on the benefits of laparoscopic surgery for patients who are obese and those who are elderly, its benefits in patients who are both elderly and obese have not been clarified. In this study, we investigated the benefit of laparoscopic surgery for elderly obese patients, who are considered to be at even higher surgical risk than those who are obese or elderly. Laparoscopic surgery tended to reduce postoperative complications without worsening the quality of surgery or the prognosis in elderly obese patients when compared with nonelderly obese patients. These findings suggest that laparoscopic surgery is a safe and beneficial treatment option for colon cancer in elderly obese patients.

The main strength of this study is that we were able to collect cases from many hospitals specializing in colorectal cancer surgery, which we believe eliminated any effect of bias introduced by the experience of the surgeon. Laparoscopic surgery is more difficult in obese patients, and studies that have failed to demonstrate a benefit of laparoscopic surgery in these patients have acknowledged the problem of variable surgeon proficiency.^30^ Furthermore, we accounted for important clinical factors, including hypertension, diabetes, and cardiovascular, respiratory, and cerebral diseases, which could have influenced our results. The main limitation of this research is that the study population was Japanese, and the definition of obesity differs between Japan (BMI ≥25) and the West (BMI ≥30).^31^ In addition, the results of this study are only applicable to patients with special backgrounds because only obese patients were included in this study. Therefore, the generalizability our findings to other populations may be limited.

## 5 CONCLUSION

Laparoscopic surgery may be safely performed for colon cancer in elderly obese patients without worsening their prognosis. There is no difference in the effectiveness of laparoscopic surgery for colon cancer between nonelderly and elderly obese patients.

## FUNDING INFORMATION

This study was funded by the Japan Society of Clinical Oncology and the Japanese Foundation for Research and Promotion of Endoscopy.

## CONFLICT OF INTEREST STATEMENT

Masafumi Inomata is an editorial member. The other authors declare no conflicts of interest for this article.

## ETHICAL STATEMENTS

The protocol for this research project was approved by the Ethics Committee of Kyoto University (Approval No. R1728).

## Supporting information


Figure S1.
