# Supplementary figures and images for: Impact of laparoscopic surgery on short‐term and long‐term outcomes in elderly obese patients with colon cancer
Source: Ann Gastroenterol Surg. 2023 Apr 23;7(5):757–64. doi: 10.1002/ags3.12678 (PMC10472405; doi:10.1002/ags3.12678)

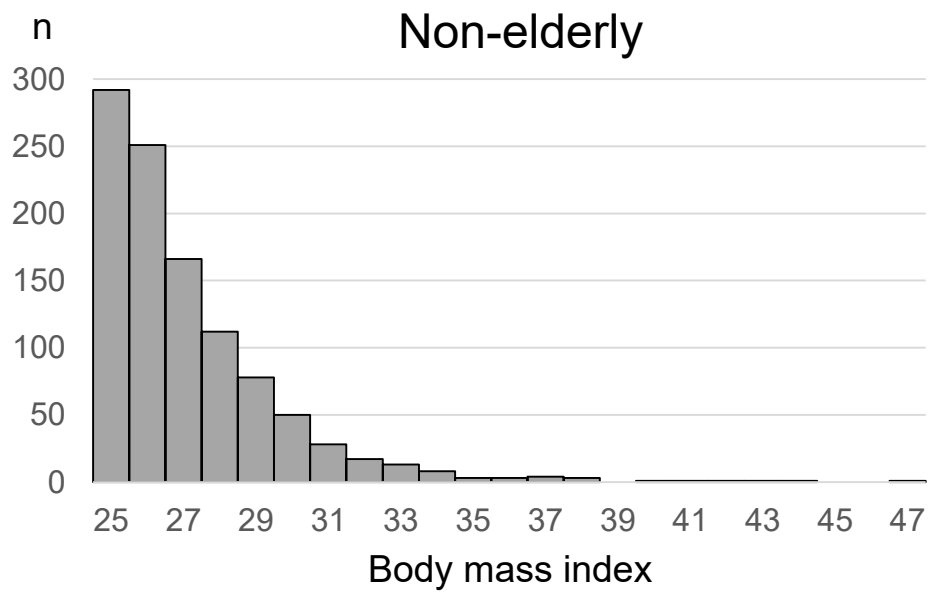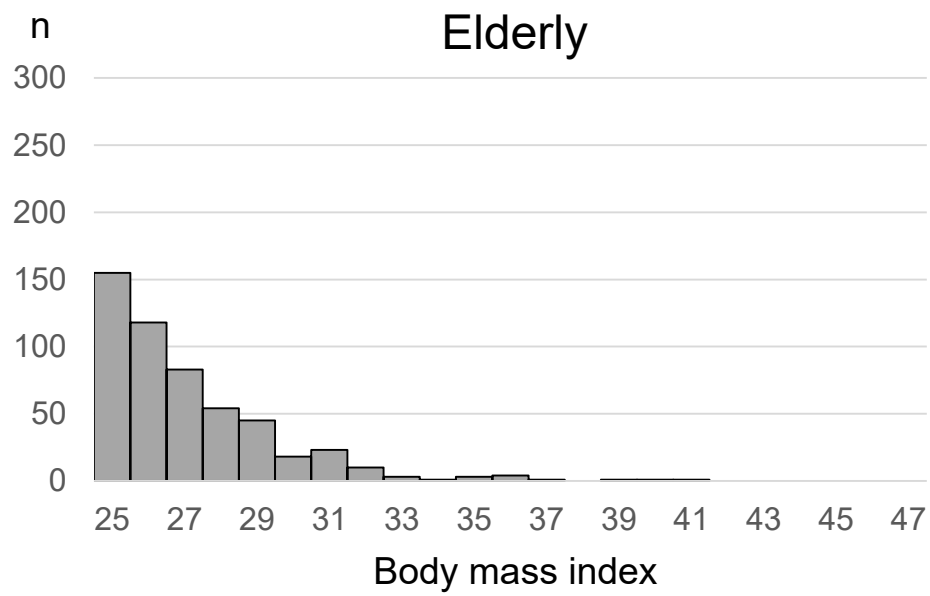

Supplement: Supplementary file 1 — Figure S1. [file AGS3-7-757-s001.pdf]
